# Supplementary material for: A high rate of COVID-19 vaccine hesitancy in a large-scale survey on Arabs
Source: eLife. 2021 May 27;10:e68038. doi: 10.7554/eLife.68038 (PMC8205489; doi:10.7554/eLife.68038)
Supplement: Supplementary file 1. [file elife-68038-supp1.pdf]

Survey of the attitudes of Arabs towards COVID-19 Vaccines

This survey aims to identify the degree of acceptance by Arabs of the COVID-19 vaccines and the barriers against this acceptance. The survey is conducted by a research group composed of a medical doctor consultant in oncology and two professors in the field of pharmacology.

1. I, the survey participant, acknowledge that the purpose of this survey and the identity of the party conducting it were made clear to me. I willfully and voluntarily participate in this survey. I understand that my personal information will remain confidential and that the survey results will be displayed anonymously for the purpose of scientific research only.

   ☐ I Agree
   ☐ I Disagree. I would like to withdraw from the survey

2. Country you currently live in

   List of All Countries

3. Gender

   ☐ Male
   ☐ Female

4. Age

_______________ years

5. Do you have any chronic disease:

   ☐ No, I don’t have any chronic conditions
   ☐ Diabetes
   ☐ Hypertension
   ☐ Heart disease
   ☐ Cancer
   ☐ Chronic bronchitis/ Chronic Obstructive Pulmonary Disease
   ☐ Other. Please specify:

   ____

6. Have you (or do you suspect you have) been infected with COVID-19?

   ☐ Yes

☐ No
☐ Not sure

7. Was the infection confirmed with a COVID-19 lab test?
☐ Yes, and got a positive result
☐ Yes, and got a negative result
☐ No, I have not tested

8. What is your highest academic achievement?
☐ Less than high school diploma (e.g. a student in school)
☐ High school diploma
☐ Some education with no degree yet (e.g. college student, community college student)
☐ University degree (Bachelor or equivalent) or Diploma
☐ A postgraduate degree (masters, PhD, or equivalents like MD fellowship)

9. What is the nature of your occupation?
☐ I am a healthcare worker
☐ I am not a healthcare worker

10. Do you take the flu vaccine yearly?
☐ Yes, every year
☐ Yes, some of the years
☐ Rarely
☐ Never

11. Do you know the types of vaccines officially approved by your country — for which you will answer the rest of questions below?
Whenever the word "vaccine" appears in subsequent questions, it will apply to the vaccine type(s) you choose here, or to vaccines in general.
☐ I don't know
☐ The American vaccine from Pfizer/BioNTech or Moderna
☐ The European vaccine from Oxford-AstraZeneca
☐ The Chinese vaccine from Sinopharm or Sinovac

☐ The Russian Vaccine Sputnik V
☐ The Indian vaccine

12. Up to this survey’s date, have you taken the vaccine?
☐ Yes, only the first dose
☐ Yes, both doses
☐ No

13. Did you have any side effects?
☐ No, I did not
☐ Yes, I had mild to moderate side effects
☐ Yes, I had severe side effects
- Mention them ________________________

14. Do you intend to take the vaccine if the option is available to you?
☐ Yes
☐ No
☐ Not sure
☐ Depends on the type of vaccine
☐ I will wait and see its effects on others

15. In your opinion, what is the best way to deal with the vaccine in your country?
☐ To let people choose if they want to take it or not
☐ To mandate it on populations in which the vaccine was proven to be effective and safe as per clinical studies
☐ To mandate it on certain categories of people
☐ To give work and transportation privileges to whomever takes the vaccine
☐ To not give it to anyone
☐ I don’t know

16. In your opinion, to what extent do others in your country need the vaccine?
☐ No one needs it
☐ Specific categories of people need it, but they're not the

majority

☐ It is needed for whomever the vaccine was proven to be effective and safe as per clinical studies

☐ I don't know

17. In case you are opposed to or hesitant about taking the vaccine, or even if you have decided to take it but have some reservations (not enough to prevent you from taking it eventually), what is/are the reason(s) for your hesitation or reservations?

Notes:
1. Please choose all reasons that apply to you.
2. The statements below do not represent the researchers' opinion; but rather the reasons for reluctance shared among the public

☐ I do not have any reservations about taking the vaccine

☐ I have been/ still am infected with Coronavirus and thus do not need the vaccine

☐ I am afraid side effects of the vaccine will develop, other than what has been disclosed

☐ I am not an eligible vaccine candidate; I am younger than 16 or I am pregnant/nursing.

☐ No need for the vaccine as rates of viral infection are decreasing

☐ The vaccine production has been rushed, making me doubt the credibility of the producing company

☐ No need for the vaccine as most people in my country have already been infected

☐ No need for the vaccine as the vast majority of infected people recover

☐ I don't think I will get infected with the virus

☐ I heard that the vaccine contains Aluminum which could harm the brain

☐ I do not believe in vaccines in general

☐ Coronavirus is a conspiracy and the vaccine is part of this conspiracy

☐ There are no published studies on the vaccine

☐ The vaccine has not been tested on a large enough number of people, just tens or hundreds

☐ I do not trust the published studies, nor the company producing the vaccine

☐ I think that the Coronavirus pandemic is over-exaggerated to benefit the pharmaceutical companies producing the vaccine. The vaccine is ineffective in preventing the COVID-19 infection

☐ The vaccine might lose its efficacy against the new viral strains

☐ I know/heard of people who took the vaccine but still got infected with Covid-19 later on

☐ I believe/I fear that the immunity gained from the vaccine does not last long

☐ I have heard about deaths related to the vaccine

☐ I have severe allergies to some foods or drugs

☐ I heard that the majority of people who took the vaccine had side effects such as general fatigue, muscle and joint pain, headaches and fever

☐ I do not want to suffer the side effects mentioned in the studies

☐ I am afraid the vaccine will irreversibly alter my DNA causing lifelong effects

☐ I have chronic diseases and thus fear to be harmed by the vaccine

☐ I believe the vaccine itself can cause the disease

☐ I don’t like needles

☐ The vaccine was not tested in Arabs and the results of the studies are not necessarily applicable to other races

☐ Not enough time has passed to verify the vaccine’s safety

☐ I do not trust the healthcare policies applied in my country
