# Supplementary material for: A high rate of COVID-19 vaccine hesitancy in a large-scale survey on Arabs
Source: eLife. 2021 May 27;10:e68038. doi: 10.7554/eLife.68038 (PMC8205489; doi:10.7554/eLife.68038)
Supplement: Supplementary file 2. [file elife-68038-supp2.pdf]

Table 1 supplementary. Differences in Barriers for Acceptance according to Country of Residence, Gender and Academic Achievement, compared using chi-square test.

Question	Country			Gender			Academic Education		
	Arab countries	Other countries	p	Female	Male	p	Higher education	Lower education	p
	25884 (84.1)	4895 (15.9)		12804 (41.6)	17975 (58.4)		18942 (61.5)	11837 (38.5)	
**I had/have COVID**	2820 (86.7)	434 (13.3)	NS	1271 (39.1)	1983 (60.9)	<0.0001	2124 (65.3)	1130 (34.7)	<0.001
**Afraid of unknown side effects**	17505 (83.7)	3405 (16.3)	<0.0001	8777 (42.0)	12133 (58.0)	<0.0001	13211 (63.2)	7699 (36.8)	<0.0001
**I am not eligible (pregnant or <16 old)**	841 (83.9)	161 (16.1)	<0.0001	843 (84.1)	159 (15.9)	<0.0001	591 (59.0)	411 (41.0)	<0.0001
**Infection rate decreasing**	2971 (90.9)	299 (9.1)	<0.001	1387 (42.4)	1883 (57.6)	<0.0001	2104 (64.3)	1166 (35.7)	<0.001
**Vaccine production was rushed**	13571 (84.6)	2474 (15.4)	<0.0001	6899 (43.0)	9146 (57.0)	<0.0001	10480 (65.3)	5565 (34.7)	<0.0001
**Most people already had COVID**	1925 (90.0)	215 (10.0)	<0.0001	1022 (47.8)	1118 (52.2)	<0.0001	1301 (60.8)	839 (39.2)	NS
**Most infected people recover**	6395 (83.7)	1242 (16.3)	NS	2995 (39.2)	4642 (60.8)	<0.0001	4746 (62.1)	2891 (37.9)	<0.0001
**Do not think I will get COVID19**	587 (86.3)	93 (13.7)	<0.0001	204 (30.0)	476 (70.0)	<0.0001	309 (45.4)	371 (54.6)	<0.0001
**Vaccines contain Aluminum**	2579 (83.8)	497 (16.2)	<0.0001	1583 (51.5)	1493 (48.5)	NS	1944 (63.2)	1132 (36.8)	<0.0001
**Do not believe in vaccines in general**	3681 (83.0)	756 (17.0)	<0.0001	2031 (45.8)	2406 (54.2)	<0.05	2603 (58.7)	1834 (41.3)	<0.0001
**Coronavirus/vaccine are conspiracy**	5463 (84.6)	996 (15.4)	<0.0001	2977 (46.1)	3482 (53.9)	<0.05	3851 (59.6)	2608 (40.4)	<0.0001
**No published studies on vaccine**	5991 (89.5)	702 (10.5)	<0.0001	3156 (47.2)	3537 (52.8)	NS	4305 (64.3)	2388 (35.7)	<0.05
**Insufficient numbers on studies**	8083 (86.0)	1312 (14.0)	<0.0001	4488 (47.8)	4907 (52.2)	<0.05	6072 (64.6)	3323 (35.4)	<0.001
**Do not trust company/studies**	9974 (86.0)	1617 (14.0)	<0.0001	5060 (43.7)	6531 (56.3)	<0.0001	7306 (63.0)	4285 (37.0)	<0.05
**Pandemic is exaggerated to benefit pharma**	7185 (83.5)	1420 (16.5)	<0.001	3566 (41.4)	5039 (58.6)	<0.0001	5614 (65.2)	2991 (34.8)	<0.0001

**No value for new strains**	6231 (84.1)	1176 (15.9)	<0.0001	3660 (49.4)	3747 (50.6)	<0.0001	4759 (64.3)	2648 (35.7)	NS
**May get COVID19 after Vaccine**	1240 (79.6)	318 (20.4)	<0.0001	697 (44.7)	861 (55.3)	NS	985 (63.2)	573 (36.8)	<0.05
**Vaccine immunity is short**	3507 (83.4)	700 (16.6)	<0.0001	1827 (43.4)	2380 (56.6)	<0.0001	2895 (68.8)	1312 (31.2)	<0.0001
**Vaccine may cause death**	3929 (84.4)	725 (15.6)	<0.0001	2220 (47.7)	2434 (52.3)	<0.0001	2825 (60.7)	1829 (39.3)	<0.001
**I have allergies to foods/drugs**	658 (84.5)	121 (15.5)	<0.0001	435 (55.8)	344 (44.2)	<0.05	502 (64.4)	277 (35.6)	<0.0001
**Most vaccinated people had SE**	2415 (85.9)	396 (14.1)	<0.0001	1390 (49.4)	1421 (50.6)	<0.05	1679 (59.7)	1132 (40.3)	NS
**Afraid of SE mentioned in studies**	5276 (85.6)	887 (14.4)	<0.0001	2965 (48.1)	3198 (51.9)	<0.0001	3582 (58.1)	2581 (41.9)	<0.0001
**Vaccines irreversibly alter DNA**	6388 (84.1)	1209 (15.9)	<0.0001	3464 (45.6)	4133 (54.4)	<0.05	4726 (62.2)	2871 (37.8)	<0.0001
**I have a chronic disease**	1111 (84.4)	206 (15.6)	<0.0001	653 (49.6)	664 (50.4)	<0.05	833 (63.2)	484 (36.8)	<0.0001
**Vaccine can cause COVID19**	5463 (85.5)	926 (14.5)	<0.0001	3058 (47.9)	3331 (52.1)	<0.0001	3703 (58.0)	2686 (42.0)	<0.05
**I don't like needles**	1517 (86.4)	238 (13.6)	<0.0001	965 (55.0)	790 (45.0)	<0.05	910 (51.9)	845 (48.1)	<0.0001
**Vaccines were not tested in Arabs**	2255 (87.5)	323 (12.5)	<0.0001	1264 (49.0)	1314 (51.0)	<0.001	1578 (61.2)	1000 (38.8)	<0.05
**Not enough time to test vaccine safety**	15887 (83.7)	3102 (16.3)	<0.0001	8564 (45.1)	10425 (54.9)	<0.05	12292 (64.7)	6697 (35.3)	<0.0001
**Do not trust the healthcare policies**	12373 (92.8)	959 (7.2)	<0.0001	5512 (41.3)	7820 (58.7)	<0.0001	8380 (62.9)	4952 (37.1)	<0.0001
